# Supplementary material for: Clinical and lifestyle patterns in Asian children with inflammatory bowel disease in the U.S
Source: PLoS One. 2023 Mar 22;18(3):e0281949. doi: 10.1371/journal.pone.0281949 (PMC10032481; doi:10.1371/journal.pone.0281949)
Supplement: S1 Fig — Regression models show that vitamin D levels and CRP are inversely correlated, but not for other markers of inflammation. Vitamin D levels have no significant correlation with time of the year in our cohort. (PPTX) [file pone.0281949.s001.pptx]

## Slide 1
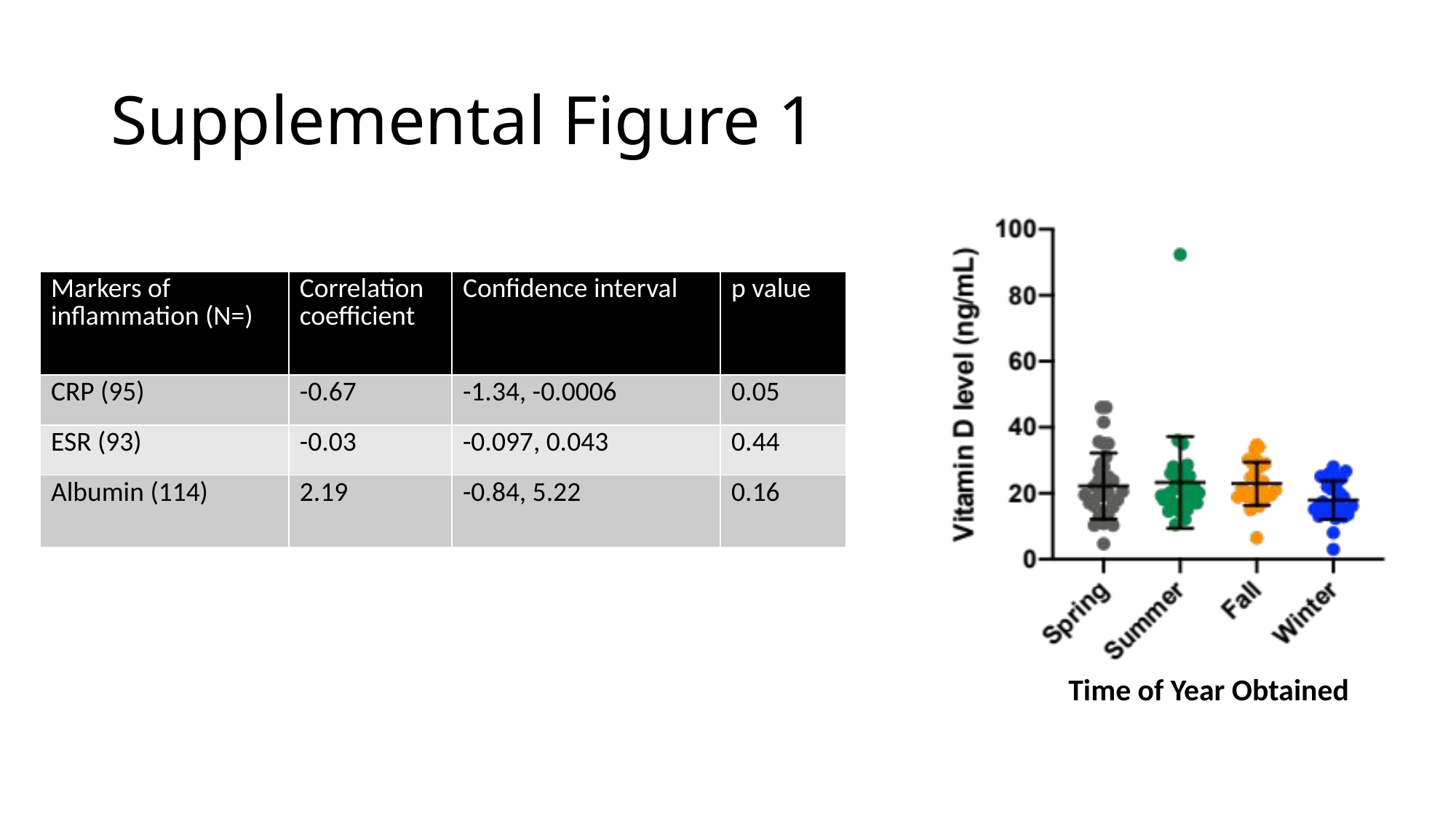

# Supplemental Figure 1
Time of Year Obtained
| Markers of inflammation (N=) | Correlation coefficient | Confidence interval | p value |
| --- | --- | --- | --- |
| CRP (95) | -0.67 | -1.34, -0.0006 | 0.05 |
| ESR (93) | -0.03 | -0.097, 0.043 | 0.44 |
| Albumin (114) | 2.19 | -0.84, 5.22 | 0.16 |
