# Supplementary material for: Clinical and lifestyle patterns in Asian children with inflammatory bowel disease in the U.S
Source: PLoS One. 2023 Mar 22;18(3):e0281949. doi: 10.1371/journal.pone.0281949 (PMC10032481; doi:10.1371/journal.pone.0281949)
Supplement: S1 Data — (DOCX) [file pone.0281949.s002.docx]

Supplemental data: IBD diagnosis codes

| ICD9 codes | Diagnosis |
| --- | --- |
| 555 | Crohn's disease |
| 555 | Regional enteritis |
| 555.0 | CD (Crohn's disease) |
| 555.0 | Crohn disease |
| 555.0 | Crohn diseases |
| 555.0 | Crohn's disease |
| 555.0 | Crohn's disease of duodenum |
| 555.0 | Crohn's disease of ileum |
| 555.0 | Crohn's disease of jejunum |
| 555.0 | Crohn's disease of pyloric antrum |
| 555.0 | Crohn's disease of pylorus |
| 555.0 | Crohn's disease of small intestine |
| 555.0 | Crohn's disease of small intestine with abscess |
| 555.0 | Crohn's disease of small intestine with complication |
| 555.0 | Crohn's disease of small intestine with fistula |
| 555.0 | Crohn's disease of small intestine with intestinal obstruction |
| 555.0 | Crohn's disease of small intestine with other complication |
| 555.0 | Crohn's disease of small intestine with rectal bleeding |
| 555.0 | Crohn's disease of small intestine without complication |
| 555.0 | Crohn's disease, small intestine |
| 555.0 | Crohn's diseases |
| 555.0 | Crohn's duodenitis |
| 555.0 | Crohn's ileitis |
| 555.0 | Crohn's jejunitis |
| 555.0 | Crohns disease |
| 555.0 | Crohns disease of small intestine |
| 555.0 | Crohns disease, small intestine |
| 555.0 | Disease crohns |
| 555.0 | Disease, Crohn |
| 555.0 | Disease, Crohn's |
| 555.0 | Disease, Crohn's intestine |
| 555.0 | Diseases, Crohn |
| 555.0 | Diseases, Crohn's |
| 555.0 | Exacerbation of Crohn's disease of small intestine |
| 555.0 | Gastroduodenal Crohn's disease |
| 555.0 | Inflammatory bowel disease, regional enteritis |
| 555.0 | Regional enteritis (Crohn's disease) |
| 555.1 | CC (Crohn's colitis) |
| 555.1 | CD (Crohn's disease) |
| 555.1 | Cicatrizing enterocolitis |
| 555.1 | Colitides, granulomatous |
| 555.1 | Colitis (granulomatous) |
| 555.1 | Colitis, granulomatous |
| 555.1 | Colitis, regional |
| 555.1 | Crohn disease |
| 555.1 | Crohn diseases |
| 555.1 | Crohn's colitis |
| 555.1 | Crohn's disease |
| 555.1 | Crohn's disease of colon |
| 555.1 | Crohn's disease of large bowel |
| 555.1 | Crohn's disease of large intestine |
| 555.1 | Crohn's disease of large intestine with abscess |
| 555.1 | Crohn's disease of large intestine with complication |
| 555.1 | Crohn's disease of large intestine with fistula |
| 555.1 | Crohn's disease of large intestine with intestinal obstruction |
| 555.1 | Crohn's disease of large intestine with other complication |
| 555.1 | Crohn's disease of large intestine with rectal bleeding |
| 555.1 | Crohn's disease of large intestine without complication |
| 555.1 | Crohn's disease of perianal region |
| 555.1 | Crohn's disease of rectum |
| 555.1 | Crohn's disease of the colon |
| 555.1 | Crohn's disease, colon |
| 555.1 | Crohn's disease, large intestine |
| 555.1 | Crohn's diseases |
| 555.1 | Crohn's proctitis |
| 555.1 | Crohns disease |
| 555.1 | Crohns disease of colon |
| 555.1 | Crohns disease, colon |
| 555.1 | Crohns disease, large intestine |
| 555.1 | Disease, Crohn |
| 555.1 | Disease, Crohn's |
| 555.1 | Disease, Crohn's intestine |
| 555.1 | Diseases, Crohn |
| 555.1 | Diseases, Crohn's |
| 555.1 | Eleocolitis |
| 555.1 | Enteritides, granulomatous |
| 555.1 | Enteritides, regional |
| 555.1 | Enteritis, granulomatous |
| 555.1 | Enteritis, regional |
| 555.1 | Enteritis, regional of |
| 555.1 | GC (granulomatous colitis) |
| 555.1 | Granulmatous colitis |
| 555.1 | Granulomatous colitides |
| 555.1 | Granulomatous colitis |
| 555.1 | Granulomatous enteritides |
| 555.1 | Granulomatous enteritis |
| 555.1 | Granulomatous proctitis |
| 555.1 | Ileitides, regional |
| 555.1 | Ileitides, terminal |
| 555.1 | Ileitis, regional |
| 555.1 | Ileitis, terminal |
| 555.1 | Ileocolitides |
| 555.1 | Ileocolitis |
| 555.1 | Inflammatory bowel disease, regional enteritis |
| 555.1 | Perianal Crohn's disease |
| 555.1 | Reg enteritis, lg intest |
| 555.1 | Regional colitis |
| 555.1 | Regional enteritides |
| 555.1 | Regional enteritis |
| 555.1 | Regional enteritis (Crohn's disease) |
| 555.1 | Regional enteritis of colon |
| 555.1 | Regional enteritis of large bowel |
| 555.1 | Regional enteritis of large intestine |
| 555.1 | Regional enteritis of rectum |
| 555.1 | Regional enteritis of the rectum |
| 555.1 | Regional enteritis of unspecified site |
| 555.1 | Regional enteritis-rectum |
| 555.1 | Regional enterocolitis |
| 555.1 | Regional ileitides |
| 555.1 | Regional ileitis |
| 555.1 | Segmental colitis |
| 555.1 | Terminal ileitides |
| 555.1 | Terminal ileitis |
| 555.1 | Transmural colitis |
| 555.2 | Crohn's disease of both small and large intestine with abscess |
| 555.2 | Crohn's disease of both small and large intestine with complication |
| 555.2 | Crohn's disease of both small and large intestine with fistula |
| 555.2 | Crohn's disease of both small and large intestine with intestinal obstruction |
| 555.2 | Crohn's disease of both small and large intestine with other complication |
| 555.2 | Crohn's disease of both small and large intestine with rectal bleeding |
| 555.2 | Crohn's disease of both small and large intestine without complication |
| 555.2 | Crohn's disease of small and large intestines |
| 555.2 | Crohn's disease, small and large intestine |
| 555.2 | Crohn's ileocolitis |
| 555.2 | Granulomatous enterocolitis |
| 555.2 | Granulomatous ileocolitis |
| 555.2 | Ileocolitis, regional |
| 555.2 | Reg enterit sm/lg intest |
| 555.2 | Regional enteritis of small intestine with large intestine |
| 555.2 | Regional ileocolitis |
| 555.9 | Acute Crohn's disease |
| 555.9 | Arthritis in Crohn's disease |
| 555.9 | Arthropathy in Crohn's disease |
| 555.9 | Arthropathy in Crohn's disease (regional enteritis) |
| 555.9 | Arthropathy in regional enteritis |
| 555.9 | CD (Crohn's disease) |
| 555.9 | Cicatrising enterocolitis |
| 555.9 | Cicatrizing enterocolitis |
| 555.9 | Crohn disease |
| 555.9 | Crohn diseases |
| 555.9 | Crohn's |
| 555.9 | Crohn's disease |
| 555.9 | Crohn's disease (regional enteritis) |
| 555.9 | Crohn's disease of intestine |
| 555.9 | Crohn's disease unspecified |
| 555.9 | Crohn's disease with abscess |
| 555.9 | Crohn's disease with complication |
| 555.9 | Crohn's disease with fistula |
| 555.9 | Crohn's disease with intestinal obstruction |
| 555.9 | Crohn's disease with other complication |
| 555.9 | Crohn's disease with rectal bleeding |
| 555.9 | Crohn's disease without complication |
| 555.9 | Crohn's disease, acute |
| 555.9 | Crohn's disease, unspecified |
| 555.9 | Crohn's diseases |
| 555.9 | Crohn's regional enteritis |
| 555.9 | Crohn's related arthritis |
| 555.9 | Crohns disease |
| 555.9 | Disease crohns |
| 555.9 | Disease, Crohn |
| 555.9 | Disease, Crohn's |
| 555.9 | Disease, Crohn's intestine |
| 555.9 | Diseases, Crohn |
| 555.9 | Diseases, Crohn's |
| 555.9 | Eleocolitis |
| 555.9 | Enteritides, granulomatous |
| 555.9 | Enteritides, regional |
| 555.9 | Enteritis (regional) |
| 555.9 | Enteritis, granulomatous |
| 555.9 | Enteritis, regional |
| 555.9 | Enteritis, regional of |
| 555.9 | Exacerbation of Crohn's disease |
| 555.9 | Granulomatous colitides |
| 555.9 | Granulomatous enteritides |
| 555.9 | Granulomatous enteritis |
| 555.9 | Ileitides, regional |
| 555.9 | Ileitides, terminal |
| 555.9 | Ileitis, regional |
| 555.9 | Ileitis, terminal |
| 555.9 | Ileocolitides |
| 555.9 | Ileocolitis |
| 555.9 | Inflammatory bowel disease (Crohn's disease) |
| 555.9 | Inflammatory bowel disease, regional enteritis |
| 555.9 | Juvenile arthritis in Crohn's disease |
| 555.9 | Juvenile arthritis in Crohn's disease (regional enteritis) |
| 555.9 | Peripheral arthropathy due to Crohn's disease |
| 555.9 | Peripheral arthropathy of due to Crohn's disease |
| 555.9 | RE (regional enteritis) |
| 555.9 | Regional colitis |
| 555.9 | Regional enteritides |
| 555.9 | Regional enteritis |
| 555.9 | Regional enteritis (Crohn's disease) |
| 555.9 | Regional enteritis of unspecified site |
| 555.9 | Regional enterocolitis |
| 555.9 | Regional ileitides |
| 555.9 | Regional ileitis |
| 555.9 | Terminal ileitides |
| 555.9 | Terminal ileitis |
| 556 | Colitides, ulcerative |
| 556 | Colitis ulcerative |
| 556 | Colitis, chronic, ulcerative |
| 556 | Colitis, ulcerative |
| 556 | Colitis, ulcerative chronic |
| 556 | Idiopathic proctocolitis |
| 556 | Megacolon toxic |
| 556 | Megacolon, toxic |
| 556 | Megacolons, toxic |
| 556 | Proctitis ulcerative |
| 556 | Proctitis, ulcerative |
| 556 | Proctocolitis, idiopathic |
| 556 | Toxic megacolon |
| 556 | Toxic megacolons |
| 556 | Ulcerative colitides |
| 556 | Ulcerative colitis |
| 556 | Ulcerative proctitis |
| 556 | Ulcerative, proctitis |
| 556.1 | Chronic ulceration of the small intestine and colon |
| 556.1 | Chronic ulcerative ileocolitis |
| 556.1 | Ulcerative (chronic) ileocolitis |
| 556.1 | Ulcerative ileocolitis |
| 556.2 | Chronic ulcerative proctitis |
| 556.2 | Chronic ulcerative proctitis with abscess |
| 556.2 | Chronic ulcerative proctitis with complication |
| 556.2 | Chronic ulcerative proctitis with fistula |
| 556.2 | Chronic ulcerative proctitis with intestinal obstruction |
| 556.2 | Chronic ulcerative proctitis with other complication |
| 556.2 | Chronic ulcerative proctitis with rectal bleeding |
| 556.2 | Chronic ulcerative proctitis without complications |
| 556.2 | Coloproctitis |
| 556.2 | Colorectitis |
| 556.2 | Hemorrhagic proctocolitides |
| 556.2 | Hemorrhagic proctocolitis |
| 556.2 | Hemorrhagic rectocolitides |
| 556.2 | Hemorrhagic rectocolitis |
| 556.2 | IP (idiopathic proctitis) |
| 556.2 | Idiopathic proctitis |
| 556.2 | Idiopathic proctocolitis |
| 556.2 | Nonspecific ulcerative proctitis |
| 556.2 | Proctitis ulcerative |
| 556.2 | Proctitis, idiopathic |
| 556.2 | Proctitis, ulcerative |
| 556.2 | Proctitis, ulcerative chronic |
| 556.2 | Proctocolitides |
| 556.2 | Proctocolitides, hemorrhagic |
| 556.2 | Proctocolitides, ulcerative |
| 556.2 | Proctocolitis |
| 556.2 | Proctocolitis, haemorrhagic |
| 556.2 | Proctocolitis, hemorrhagic |
| 556.2 | Proctocolitis, idiopathic |
| 556.2 | Proctocolitis, ulcerative |
| 556.2 | Rectocolitides |
| 556.2 | Rectocolitides, hemorrhagic |
| 556.2 | Rectocolitides, ulcerative |
| 556.2 | Rectocolitis |
| 556.2 | Rectocolitis, haemorrhagic |
| 556.2 | Rectocolitis, hemorrhagic |
| 556.2 | Rectocolitis, ulcerative |
| 556.2 | UC (ulcerative colitis confined to rectum) |
| 556.2 | Ulcerative (chronic) proctitis |
| 556.2 | Ulcerative chronic proctitis |
| 556.2 | Ulcerative colitis confined to rectum |
| 556.2 | Ulcerative proctitis |
| 556.2 | Ulcerative proctitis, chronic |
| 556.2 | Ulcerative proctitis, nonspecific |
| 556.2 | Ulcerative proctocolitides |
| 556.2 | Ulcerative proctocolitis |
| 556.2 | Ulcerative rectocolitides |
| 556.2 | Ulcerative rectocolitis |
| 556.2 | Ulcerative, proctitis |
| 556.3 | Chronic ulceration of the rectum and the colon |
| 556.3 | Chronic ulcerative proctosigmoiditis |
| 556.3 | Chronic ulcerative rectosigmoiditis |
| 556.3 | Chronic ulcerative rectosigmoiditis with abscess |
| 556.3 | Chronic ulcerative rectosigmoiditis with complication |
| 556.3 | Chronic ulcerative rectosigmoiditis with fistula |
| 556.3 | Chronic ulcerative rectosigmoiditis with intestinal obstruction |
| 556.3 | Chronic ulcerative rectosigmoiditis with other complication |
| 556.3 | Chronic ulcerative rectosigmoiditis with rectal bleeding |
| 556.3 | Chronic ulcerative rectosigmoiditis without complications |
| 556.3 | Proctosigmoiditis, ulcerative |
| 556.3 | Proctosigmoiditis, ulcerative chronic |
| 556.3 | Rectosigmoiditis, ulcerative |
| 556.3 | Ulcerative (chronic) proctosigmoiditis |
| 556.3 | Ulcerative (chronic) rectosigmoiditis |
| 556.3 | Ulcerative (chronic) rectosigmoiditis with unspecified complications |
| 556.3 | Ulcerative chronic rectosigmoiditis |
| 556.3 | Ulcerative colitis confined to rectum and sigmoid colon |
| 556.3 | Ulcerative colitis, rectosigmoid |
| 556.3 | Ulcerative proctosigmoiditis |
| 556.3 | Ulcerative rectosigmoiditis |
| 556.5 | Chronic left-sided ulcerative colitis |
| 556.5 | Inflammatory bowel disease (ulcerative colitis) |
| 556.5 | Left sided colitis with abscess |
| 556.5 | Left sided colitis with fistula |
| 556.5 | Left sided colitis with intestinal obstruction |
| 556.5 | Left sided colitis with other complication |
| 556.5 | Left sided colitis with rectal bleeding |
| 556.5 | Left sided colitis without complications |
| 556.5 | Left sided ulcerative (chronic) colitis |
| 556.5 | Left sided ulcerative colitis |
| 556.5 | Left-sided ulcerative (chronic) colitis |
| 556.5 | Left-sided ulcerative colitis |
| 556.5 | Ulcerative colitis, left sided |
| 556.5 | Ulcerative colitis, left sided, chronic |
| 556.6 | Chronic ulcerative pancolitis |
| 556.6 | Pancolitis |
| 556.6 | Ulcerative chronic pancolitis |
| 556.6 | Ulcerative chronic pancolitis with abscess |
| 556.6 | Ulcerative chronic pancolitis with fistula |
| 556.6 | Ulcerative chronic pancolitis with intestinal obstruction |
| 556.6 | Ulcerative chronic pancolitis with other complication |
| 556.6 | Ulcerative chronic pancolitis with rectal bleeding |
| 556.6 | Ulcerative chronic pancolitis without complications |
| 556.6 | Ulcerative colitis, universal |
| 556.6 | Ulcerative pancolitis |
| 556.6 | Universal ulcerative (chronic) colitis(556.6) |
| 556.6 | Universal ulcerative colitis |
| 556.6 | Universal ulceritive colitis |
| 556.8 | Eosinophilic ulcerative colitis |
| 556.8 | Other ulcerative colitis |
| 556.8 | Other ulcerative colitis with abscess |
| 556.8 | Other ulcerative colitis with complication |
| 556.8 | Other ulcerative colitis with fistula |
| 556.8 | Other ulcerative colitis with intestinal obstruction |
| 556.8 | Other ulcerative colitis with other complication |
| 556.8 | Other ulcerative colitis with rectal bleeding |
| 556.8 | Other ulcerative colitis without complication |
| 556.8 | Ulcerative colitis |
| 556.8 | Ulcerative colitis NEC |
| 556.8 | Ulcerative colitis with abscess |
| 556.8 | Ulcerative colitis with fistula |
| 556.8 | Ulcerative colitis with intestinal obstruction |
| 556.8 | Ulcerative colitis with other complication |
| 556.8 | Ulcerative colitis with rectal bleeding |
| 556.9 | Acute megacolon |
| 556.9 | Acute ulcerative colitis |
| 556.9 | Acute ulcerative gastroenteritis complicating pneumonia |
| 556.9 | Arthropathy in ulcerative colitis |
| 556.9 | Asymptomatic ulcerative colitis |
| 556.9 | Chronic ulcerative colitis |
| 556.9 | Colitides, ulcerative |
| 556.9 | Colitis ulcerative |
| 556.9 | Colitis, chronic, ulcerative |
| 556.9 | Colitis, ulcerative |
| 556.9 | Colitis, ulcerative chronic |
| 556.9 | Exacerbation of ulcerative colitis |
| 556.9 | Fulminant ulcerative colitis |
| 556.9 | Hemorrhagic proctocolitides |
| 556.9 | Hemorrhagic proctocolitis |
| 556.9 | Hemorrhagic rectocolitides |
| 556.9 | Hemorrhagic rectocolitis |
| 556.9 | Idiopathic ulcerative colitis |
| 556.9 | Inflammatory bowel disease (ulcerative colitis) |
| 556.9 | Juvenile arthritis in ulcerative colitis |
| 556.9 | Megacolon toxic |
| 556.9 | Megacolon, toxic |
| 556.9 | Megacolons, toxic |
| 556.9 | Mild chronic ulcerative colitis |
| 556.9 | Moderate chronic ulcerative colitis |
| 556.9 | Nonspecific ulcerative colitis |
| 556.9 | Peripheral arthropathy due to ulcerative colitis |
| 556.9 | Proctocolitides, hemorrhagic |
| 556.9 | Proctocolitides, ulcerative |
| 556.9 | Proctocolitis, hemorrhagic |
| 556.9 | Proctocolitis, ulcerative |
| 556.9 | Quiescent ulcerative colitis |
| 556.9 | Rectocolitides, hemorrhagic |
| 556.9 | Rectocolitides, ulcerative |
| 556.9 | Rectocolitis, hemorrhagic |
| 556.9 | Rectocolitis, ulcerative |
| 556.9 | Severe chronic ulcerative colitis |
| 556.9 | Toxic dilatation of colon |
| 556.9 | Toxic dilation of the colon |
| 556.9 | Toxic megacolon |
| 556.9 | Toxic megacolons |
| 556.9 | UC (ulcerative colitis) |
| 556.9 | Ulcerated colon |
| 556.9 | Ulcerative colitides |
| 556.9 | Ulcerative colitis |
| 556.9 | Ulcerative colitis unspecified |
| 556.9 | Ulcerative colitis without complications |
| 556.9 | Ulcerative colitis, acute |
| 556.9 | Ulcerative colitis, chronic |
| 556.9 | Ulcerative colitis, quiescent |
| 556.9 | Ulcerative colitis, unspecified |
| 556.9 | Ulcerative colitis, unspecified with unspecified complications |
| 556.9 | Ulcerative enteritis |
| 556.9 | Ulcerative proctocolitides |
| 556.9 | Ulcerative proctocolitis |
| 556.9 | Ulcerative rectocolitides |
| 556.9 | Ulcerative rectocolitis |
| 556.9 | Unspecified ulcerative colitis |
| 558.9 | Acute and chronic colitis |
| 558.9 | Bowel disease, inflammatory |
| 558.9 | Bowel diseases, inflammatory |
| 558.9 | CC (collagenous colitis) |
| 558.9 | Chronic colitis |
| 558.9 | Chronic enteritis |
| 558.9 | Chronic inflammation of colon |
| 558.9 | Chronic inflammatory small bowel disease |
| 558.9 | Colitis, collagenous |
| 558.9 | Colitis, indeterminate |
| 558.9 | Collagenous colitis |
| 558.9 | Disease, inflammatory bowel |
| 558.9 | Disuse colitis |
| 558.9 | Diversion colitis |
| 558.9 | IBD (inflammatory bowel disease) |
| 558.9 | Idiopathic chronic inflammatory bowel disease |
| 558.9 | Indeterminate colitis |
| 558.9 | Inflammation of colonic mucosa |
| 558.9 | Inflammation of intestines |
| 558.9 | Inflammation of large intestine |
| 558.9 | Inflammation of small intestine |
| 558.9 | Inflammation of small intestine and colon |
| 558.9 | Inflammation of the colon |
| 558.9 | Inflammation of the stomach and intestines |
| 558.9 | Inflammatory bowel disease |
| 558.9 | Inflammatory bowel disease in pediatric patient |
| 558.9 | Inflammatory bowel diseases |
| 558.9 | Inflammatory bowel diseases (IBD) |
| 558.9 | Inflammatory bowel syndrome |
| 558.9 | Neuropathy due to inflammatory bowel disease |
| 569.71 | Ileal pouchitis |
| 569.71 | Inflammation of internal ileoanal pouch |
| 569.71 | Pouchitis |
| 713.1 | Arthritis in Crohn's disease |
| 713.1 | Arthropathy in Crohn's disease |
| 713.1 | Arthropathy in Crohn's disease (regional enteritis) |
| 713.1 | Arthropathy in ulcerative colitis |
| 713.1 | Crohn's related arthritis |
| 713.1 | Peripheral arthropathy due to Crohn's disease |
| 713.1 | Peripheral arthropathy due to ulcerative colitis |
| 713.1 | Peripheral arthropathy of due to Crohn's disease |
| 714.30 | Juvenile arthritis in Crohn's disease |
| 714.30 | Juvenile arthritis in Crohn's disease (regional enteritis) |
| 714.30 | Juvenile arthritis in ulcerative colitis |
| 721.90 | Spondylitic arthritis in ulcerative colitis |
| IMO0001 | Crohn's disease of vulva |
| K50 | Crohn's disease (regional enteritis) |
| K50.0 | Crohn's disease of small intestine |
| K50.00 | Crohn's disease of small intestine without complications |
| K50.01 | Crohn's disease of small intestine with complications |
| K50.011 | Crohn's disease of small intestine with rectal bleeding |
| K50.012 | Crohn's disease of small intestine with intestinal obstruction |
| K50.013 | Crohn's disease of small intestine with fistula |
| K50.014 | Crohn's disease of small intestine with abscess |
| K50.018 | Crohn's disease of small intestine with other complication |
| K50.019 | Crohn's disease of small intestine with unspecified complications |
| K50.1 | Crohn's disease of large intestine |
| K50.10 | Crohn's disease of large intestine without complications |
| K50.11 | Crohn's disease of large intestine with complications |
| K50.111 | Crohn's disease of large intestine with rectal bleeding |
| K50.112 | Crohn's disease of large intestine with intestinal obstruction |
| K50.113 | Crohn's disease of large intestine with fistula |
| K50.114 | Crohn's disease of large intestine with abscess |
| K50.118 | Crohn's disease of large intestine with other complication |
| K50.119 | Crohn's disease of large intestine with unspecified complications |
| K50.8 | Crohn's disease of both small and large intestine |
| K50.80 | Crohn's disease of both small and large intestine without complications |
| K50.81 | Crohn's disease of both small and large intestine with complications |
| K50.811 | Crohn's disease of both small and large intestine with rectal bleeding |
| K50.812 | Crohn's disease of both small and large intestine with intestinal obstruction |
| K50.813 | Crohn's disease of both small and large intestine with fistula |
| K50.814 | Crohn's disease of both small and large intestine with abscess |
| K50.818 | Crohn's disease of both small and large intestine with other complication |
| K50.819 | Crohn's disease of both small and large intestine with unspecified complications |
| K50.9 | Crohn's disease, unspecified |
| K50.90 | Crohn's disease, unspecified, without complications |
| K50.91 | Crohn's disease, unspecified, with complications |
| K50.911 | Crohn's disease, unspecified, with rectal bleeding |
| K50.912 | Crohn's disease, unspecified, with intestinal obstruction |
| K50.913 | Crohn's disease, unspecified, with fistula |
| K50.914 | Crohn's disease, unspecified, with abscess |
| K50.918 | Crohn's disease, unspecified, with other complication |
| K50.919 | Crohn's disease, unspecified, with unspecified complications |
| K51 | Ulcerative colitis |
| K51.00 | Ulcerative (chronic) pancolitis without complications |
| K51.011 | Ulcerative (chronic) pancolitis with rectal bleeding |
| K51.014 | Ulcerative (chronic) pancolitis with abscess |
| K51.018 | Ulcerative (chronic) pancolitis with other complication |
| K51.019 | Ulcerative (chronic) pancolitis with unspecified complications |
| K51.20 | Ulcerative (chronic) proctitis without complications |
| K51.211 | Ulcerative (chronic) proctitis with rectal bleeding |
| K51.218 | Ulcerative (chronic) proctitis with other complication |
| K51.219 | Ulcerative (chronic) proctitis with unspecified complications |
| K51.30 | Ulcerative (chronic) rectosigmoiditis without complications |
| K51.311 | Ulcerative (chronic) rectosigmoiditis with rectal bleeding |
| K51.8 | Other ulcerative colitis |
| K51.80 | Other ulcerative colitis without complications |
| K51.81 | Other ulcerative colitis with complications |
| K51.811 | Other ulcerative colitis with rectal bleeding |
| K51.812 | Other ulcerative colitis with intestinal obstruction |
| K51.813 | Other ulcerative colitis with fistula |
| K51.814 | Other ulcerative colitis with abscess |
| K51.818 | Other ulcerative colitis with other complication |
| K51.819 | Other ulcerative colitis with unspecified complications |
| K51.9 | Ulcerative colitis, unspecified |
| K51.90 | Ulcerative colitis, unspecified, without complications |
| K51.91 | Ulcerative colitis, unspecified, with complications |
| K51.911 | Ulcerative colitis, unspecified with rectal bleeding |
| K51.912 | Ulcerative colitis, unspecified with intestinal obstruction |
| K51.913 | Ulcerative colitis, unspecified with fistula |
| K51.914 | Ulcerative colitis, unspecified with abscess |
| K51.918 | Ulcerative colitis, unspecified with other complication |
| K51.919 | Ulcerative colitis, unspecified with unspecified complications |
| K52.3 | Indeterminate colitis |
