# Supplementary material for: Clinical and lifestyle patterns in Asian children with inflammatory bowel disease in the U.S
Source: PLoS One. 2023 Mar 22;18(3):e0281949. doi: 10.1371/journal.pone.0281949 (PMC10032481; doi:10.1371/journal.pone.0281949)
Supplement: S2 Data — (PDF) [file pone.0281949.s003.pdf]

Dietary Survey

Baseline Information

Diet and Inflammatory Bowel Disease in Asian Children
The following survey has 6 sections and takes 10 minutes to complete.

Patient's name: [first_name] [last_name]

What is [first_name]'s race?

- ☐ American Indian/Alaskan
- ☐ Asian (including Indian)
- ☐ Black/African American
- ☐ White
- ☐ Hispanic
- ☐ Native Hawaiian or Other Pacific Islander
- ☐ Other

(Check all that apply)

Specify race

____

What is [first_name] family's country of origin?

- ☐ India
- ☐ Pakistan
- ☐ Bangladesh
- ☐ Sri Lanka
- ☐ China
- ☐ Korea
- ☐ Others

(Check all that apply.)

Specify country of origin

____

What region of the country did [first_name]'s family originate from? (city/province)

____

Is [first_name] born in the U.S.?

- ◯ Yes
- ◯ No

At what age did [first_name] immigrate to the U.S.?

____

Is [first_name] a second generation immigrant? ([first_name] is born in the U.S. but parents are immigrants from another country)

- ◯ Yes
- ◯ No

What is [first_name] and family's religious affiliation? (Optional)

- ☐ Hindu
- ☐ Muslim
- ☐ Christian/Catholic
- ☐ Jain
- ☐ Sikh
- ☐ Buddhist
- ☐ Other

Specify religion

____

Is there a family history of IBD in [first_name]'s first degree relatives? (mom, dad, sister, or brother)

- ☐ None
- ☐ Ulcerative colitis
- ☐ Crohn's disease
- ☐ Indeterminate colitis

(Check all that apply)

Is there a family history of IBD in [first_name]'s second degree relatives? (grandfather, grandmother, aunt, uncle, cousin)

- ☐ None
- ☐ Ulcerative colitis
- ☐ Crohn's disease
- ☐ Indeterminate colitis

(Check all that apply)

Acculturation

Traditional breakfast (Ex: Idli, Vada, Dosa, Paratha, Upma, Roti, Gyeranjjim, Rice, Miso Soup)

Photo showing representative images of traditional breakfast items

Western breakfast (Ex: Eggs, Pancakes, Potatoes, Toast, Oatmeal, Muffins, Waffles, Cereal)

Photo showing representative images of Western breakfast items

Does [first_name] typically eat a traditional style breakfast vs Western style breakfast?

Traditional style — Both equally — Western style

(Place a mark on the scale above)

(Ex: mango pickle, lemon pickle, kimchi, natto, etc)

Photo showing representative images of traditionally preserved foods

Does [first_name] typically eat traditionally preserved foods?

None/Rarely | Few times a month | Everyday

(Place a mark on the scale above)

Does [first_name] typically eat traditional foods vs Western-style meals AT HOME?

Traditional meals | Both equally | Non-traditional meals (Western style)

(Place a mark on the scale above)

Does [first_name] typically eat traditional foods vs Western-style meals OUT OF THE HOME?

Traditional meals | Both equally | Non-traditional meals (Western style)

(Place a mark on the scale above)

(Ex: cumin, turmeric, garlic, saffron, anise, parsley, cilantro, lemongrass, etc)

Photo showing representative images of traditional spices and herbs

Does [first_name] typically eat foods that uses variety of traditional spices and herbs in cooking?

None/Rarely | Few times a month | Everyday

(Place a mark on the scale above)

If so, what kind of spices and herbs are most commonly consumed by [first_name]?

- ☐ garam masala (star anise, cumin, cinnamon, cardamom, cloves etc)
- ☐ turmeric
- ☐ cumin
- ☐ coriander
- ☐ fennel
- ☐ safron
- ☐ chili
- ☐ peppercorn/sichuan pepper
- ☐ bayleaf
- ☐ other

(Choose multiple)

Other spices

____

Does [first_name]'s family shop at ethnic (Asian, Indian, etc) grocery stores or purchase traditional cooking ingredients?

Never or occasional | Sometimes (once a month) | Every grocery trip (weekly)

(Place a mark on the scale above)

Early Life Exposure

Was [first_name] born by C-section or vaginal delivery?

◯ Vaginal ◯ C-section

Was [first_name] ever breastfed or fed breast milk?

◯ Yes ◯ No

How old was [first_name] when he/she completely stopped breast milk?

◯ Less than 6 months old
◯ 6 months to 1 year old
◯ Over 1 year old

How old was [first_name] when he/she started drinking infant formula?

◯ Never drank formula
◯ Before 6 months
◯ Between 6 months and 1 year old

How old was [first_name] when he/she completely stopped drinking formula?

◯ Before 6 months old
◯ Between 6 months and 1 year old
◯ After 1 year old

How often did [first_name] consume cow's milk products as a toddler (1-3 year old)? (milk, yogurt, cream, cheese, etc)

Rarely Few times a month Everyday

(Place a mark on the scale above)

Did [first_name] drink other kinds of milk consistently as a toddler (1-3 years old)?

☐ Soy milk
☐ Almond milk
☐ Coconut milk
☐ Other milk
(Check all that apply)

Specify other milks

____

How often did [first_name] drink [other_milk] as a toddler (1-3 years old)?

Rarely Few times a month Everyday

(Place a mark on the scale above)

Pre-diagnosis Diet (Recall pattern of eating 1-2 years prior to diagnosis)

Before diagnosis , about how many times a week did [first_name] get a complete school breakfast/lunch?

◯ Never
◯ 1-4 times a week
◯ Once everyday
◯ At least twice a day

Before diagnosis , did [first_name] ever eat prepared meals from an after-school program or other community programs?

◯ Never
◯ Few times a week
◯ Daily

Before diagnosis , how many meals did [first_name] eat that were prepared away from home in places such as restaurants, fast food chains, pizza place, food stands?

◯ None
◯ Between 1-6 times a week
◯ Once daily
◯ More than once a day

Before diagnosis , how often did [first_name] eat ready-to-eat foods from the grocery store? (Ready-to-eat foods in salad bars and deli counters. Do not include sliced meat or cheese or canned foods.)

- ◯ Never
- ◯ Between 1-6 times a week
- ◯ Once daily
- ◯ More than once a day

Before diagnosis, how often did [first_name] eat red meat?

- ◯ Never
- ◯ Less than once a month
- ◯ 1-3 times per month
- ◯ Once a week
- ◯ Several times per week
- ◯ Once or more a day

Before diagnosis , how often did [first_name] eat processed foods or foods with long expiration dates? (such as microwave dinner, pre-packaged snacks, lunchables, canned soups, etc)

Never or occasional | Few times a week | Few times a day

(Place a mark on the scale above)

One serving of each kind of fruit

Photo showing representative images of one serving of fruits

Before diagnosis, how many servings of fruits did [first_name] eat a day?

Less than 1 serving | 1 serving a day | More than 4 servings

(Place a mark on the scale above)

One serving of each kind of vegetable

Photo showing representative images of one serving of vegetables

---

Before diagnosis, how many servings of vegetables did [first_name] eat a day?

Less than 1 serving | 1 serving a day | More than 3 servings

(Place a mark on the scale above)

1 serving of fluid = 1 cup (8 fl oz/250 ml)

Photo showing representative images of one serving of sugary drinks

Before diagnosis, how much sugary drinks (soda, sweet tea, gatorade, juice, etc) did [first_name] drink a day?

Never or occasional — 1 cup a day — More than 5 cups

(Place a mark on the scale above)

One serving of dairy

Photo showing representative images of one serving of dairy

Before diagnosis, how many servings of dairy did [first_name] eat a day? (Ex: milk, paneer, khutta, yogurt, etc)

Never or occasional — one serving a day — More than 5 servings

(Place a mark on the scale above)

Before diagnosis, how many servings of foods with high fiber did [first_name] eat a day? (whole grains, vegetables, legumes, etc)

Never or occasional — 1 serving a day — More than 5 servings

(Place a mark on the scale above)

Before diagnosis, how often did [first_name] eat foods with artificial sweeteners, diet products, and food colorings? (protein bars, lean cuisine, cupcakes or cookies with food colorings, etc)

Never or occasional — Few days a week — More than once daily

(Place a mark on the scale above)

Current Diet (In the last 3 months)

In the last 30 days:
How often did [first_name] eat red meat?

- ◯ Never
- ◯ 1-3 times in a month
- ◯ Once a week
- ◯ Several times per week
- ◯ Once or more a day

In the last 30 days:
How many servings of dairy does [first_name] eat a day?

Never or occasional | 1 serving a day | More than 5 servings

(Place a mark on the scale above)

In the last 30 days:
How many servings of fruits does [first_name] eat a day?

Never or occasional | 1 serving a day | More than 5 servings

(Place a mark on the scale above)

In the last 30 days:
How many servings of vegetables does [first_name] eat a day?

Never or occasional | 1 serving a day | More than 5 servings

(Place a mark on the scale above)

In the last 30 days:
How many servings of sugary drinks (soda, sweet tea, juice, gatorade, etc) does [first_name] drink a day?

Never or occasional | 1 cup a day | More than 5 cups

(Place a mark on the scale above)

In the last 30 days:
How often does [first_name] eat processed foods or foods with long expiration dates a day?

Never or occasional | Few times a week | Few times a day

(Place a mark on the scale above)

In the last 30 days:
How many servings of foods with high fiber does [first_name] eat a day? (whole grains, vegetables, legumes, etc)

Never or occasional | 1 serving a day | More than 5 servings

(Place a mark on the scale above)

In the last 30 days:
How often does [first_name] eat foods with artificial sweeteners, diet products, and food colorings?

Never or occasional | Few days a week | More than once daily

(Place a mark on the scale above)

Supplements and Complementary/Alternative Medicine

Is [first_name] taking any supplements?

- ☐ No
- ☐ Probiotics
- ☐ Vitamins
- ☐ Fish oil
- ☐ Turmeric (supplement, not in food)
- ☐ Others

(Check all that apply)

Specify other supplements: ______________________

Which vitamins is [first_name] taking?

- ☐ Vitamin D
- ☐ Multivitamin
- ☐ Vitamin C
- ☐ Vitamin B complex
- ☐ Others

(Check all that apply)

Specify other vitamins: ______________________

Is [first_name] taking enteral therapy for IBD? (majority of calories from a formula for IBD or supplemental formula for weight gain)

- ◯ No
- ◯ Partial enteral nutrition (< 50% of daily calories from formula)
- ◯ Exclusive enteral nutrition

Is [first_name] following any diets for IBD?

- ☐ No
- ☐ SCD (specific carbohydrate diet)
- ☐ Vegetarian diet
- ☐ Gluten free diet
- ☐ Low FODMAP diet
- ☐ Other

(Check all that apply)

Specify other diet: ______________________

Is [first_name] limiting or avoiding a particular food/food groups in current diet?

- ☐ No
- ☐ Food additives (emulsifiers, coloring, etc)
- ☐ Red meat
- ☐ Dairy
- ☐ Gluten
- ☐ Fiber and roughage
- ☐ Others

(Check all that apply)

Specify other foods being avoided: ______________________

Is [first_name] using any complementary or alternative practices for [first_name]'s IBD?

- ☐ No
- ☐ Yoga/meditation
- ☐ Acupuncture
- ☐ Others

(Check all that apply)

Specify other complementary and alternative medicine used: ______________________

End

Thank you for helping us to understand the diet of children with IBD! Please move the the next page to receive your $10 Amazon gift card.

If you have any questions or comments, feel free to type them here, or email us.
