# Supplementary material for: Clinical and lifestyle patterns in Asian children with inflammatory bowel disease in the U.S
Source: PLoS One. 2023 Mar 22;18(3):e0281949. doi: 10.1371/journal.pone.0281949 (PMC10032481; doi:10.1371/journal.pone.0281949)
Supplement: S1 Table — (DOCX) [file pone.0281949.s004.docx]

**S1 Table.** Baseline characteristics of South Asian American patients.

|  | **South Asian** | | |
| --- | --- | --- | --- |
|  | Overall | Age at dx <6 yr | Male |
|  | N=108 | 19 (18%) | 63 (58%) |
| **CD** | 71 (66%) | 11 | 45 (63%) |
| **UC** | 25 (23%) | 4 | 12 (48%) |
| **IC** | 12 (11%) | 4 | 6 (50%) |
|  | | | |
| **Crohn’s disease** | | |  |
| Location, n (%) | | | N=71 |
| L1 (ileal) | | | 5 (7%) |
| L2 (colonic) | | | 18 (25%) |
| L3 (ileocolonic) | | | 46 (65%) |
| L4a (upper disease proximal to LOT) | | | 8 (11%) |
| L4b (upper disease distal to LOT) | | | 2 (3%) |
| L4ab | | | 1 (1%) |
| Behavior, n (%) | | | N=71 |
| B1 (non-stricturing, non-penetrating) | | | 57 (80%) |
| B2 (stricturing) | | | 6 (8%) |
| B3 (penetrating) | | | 2 (3%) |
| B2B3 (stricturing, penetrating) | | | 4 (6%) |
| Perianal | | | 16 (23%) |
|  | | | |
| **Ulcerative colitis** | | |  |
| Extent, n (%) | | | N=25 |
| E1 (proctitis) | | | 5 (20%) |
| E2 (left side; distal to splenic flexure) | | | 1 (4%) |
| E3 (extensive; distal to hepatic flexure) | | | 4 (16%) |
| E4 (pancolonic) | | | 15 (60%) |
